# Comparison of Methods Used to Estimate the Global Burden of Disease Related to Undernutrition and Suboptimal Breastfeeding

**DOI:** 10.1093/advances/nmy094

**Published:** 2019-04-22

**Authors:** Alexander C McLain, Edward A Frongillo, Sonja Y Hess, Ellen G Piwoz

**Affiliations:** 1 Departments of Epidemiology and Biostatistics; 2 Health Promotion, Education, and Behavior, Arnold School of Public Health, University of South Carolina, Columbia, SC; 3 Department of Nutrition, University of California, Davis, CA; 4 Bill & Melinda Gates Foundation, Seattle, WA

**Keywords:** global burden of disease, anthropometry, breastfeeding, undernutrition

## Abstract

The Global Burden of Disease study (GBD) is an ambitious effort to estimate the disease burden attributable to various risk factors. The results from the GBD are used around the world to monitor the UN established Sustainable Development Goals, set health policies and research strategies, among others. The GBD along with other studies, such as those from the Maternal Child Epidemiology Estimation Group and the Lancet Breastfeeding Series Group, produce estimates of the nutrition-related global burden of disease that exhibit considerable differences. These differences are difficult to reconcile due to the estimation methods, which in recent years have substantially increased in complexity. In this paper, we give a detailed review of the methods used by GBD and other entities to estimate the global burden of disease that is attributable to undernutrition and suboptimal breastfeeding. Further, we compare the methods to determine causes for differences in estimates. We find that the main determinant of differences in estimates is what causes of death are linked to each risk factor. Methods used to estimate nutrition-related disease burden need to be more clearly documented to foster discussion and collaboration on the important assumptions required to produce estimates.

## Introduction

Adequate nutrition to ensure healthy and productive lives is a global priority. To meet this goal, it is necessary to ensure that the scope of nutrition deficiencies is well defined and that areas struggling to achieve adequate nutrition are identified. One strategy in this effort is to estimate the global burden of deaths attributable to nutrition. The Institute for Health Metrics and Evaluation (IHME) maintains the Global Burden of Disease study (GBD), which is the main source of estimates of the global burden of disease. The GBD series started with the GBD 1990 report (1), which was commissioned by the World Bank and aimed to measure the major causes of the world's health problems. The GBD estimates for 2000, 2001, 2002, and 2004 were completed in the Disease Burden Unit of the WHO. The IHME was founded in 2007 through funding from the Bill & Melinda Gates Foundation and the State of Washington. IHME began producing GBD estimates in 2010 (2); their methods and reports were updated in 2013, 2015, and 2016 (3–5). The GBD study has increased the number of causes of death and disability from 107 for the GBD 1990 to 328 in the GBD 2016 (1,6). In the future, GBD methods and estimates will be updated annually. IHME's GBD program reports on many aspects of global health, including risk factors, causes of death, disease and injury, child and maternal mortality, and the Sustainable Development Goals. The focus here will be on the GBD risk factor estimates. Although there is overlap in the methods used for the various conditions reported by IHME, other portions of the GBD (e.g., causes of death) have unique issues not discussed here.

Other entities that have produced disease burden estimates related to nutrition include the Maternal Child Epidemiology Estimation Group (MCEE) and the Lancet Breastfeeding Series Group (LBS). MCEE produced global estimates of the impact of maternal and childhood nutrition in 2004 (7) and 2011 (8), whereas the LBS was a one-time collaboration among maternal global health experts that estimated the impact of suboptimal breastfeeding in 2015 (9). MCEE and LBS estimated the impact of suboptimal breastfeeding on the number of global fatalities with the use of the Lives Saved Tool (LiST). The LiST software was originally developed as part of the work for the Lancet Child Survival Series (10) to estimate the potential impact on global mortality of children aged <5 y of a community intervention that was universally applied. The software has since been expanded to handle different interventions, risk factors, and conditions (e.g., wasting, stunting, HIV/AIDS), and it became free and publicly available as the Spectrum software package (11). LiST can estimate the impact of >70 separate interventions on many conditions and risk factors. Studies have shown relatively good agreement between the estimates given by LiST and observed mortality reductions with different sets of interventions in different countries (12–15). The LiST software and mathematical models are periodically updated when new scientific evidence is published (15,16).

The estimates produced by GBD, MCEE, and LBS for the burden attributable to nutrition-related risk factors differ. For example, GBD 2015 estimated the impact of all causes of child and maternal malnutrition, which includes suboptimal breastfeeding, childhood undernutrition, iron deficiency, vitamin A deficiency, and zinc deficiency, as ∼2.2 million deaths in 2005 and ∼1.4 million deaths in 2015 (4). In contrast, MCEE reported the impact of the joint effects of fetal growth restriction, suboptimal breastfeeding, stunting, wasting, and vitamin A and zinc deficiencies as ∼3.1 million deaths in 2011 (8). Differences in estimates produced by these entities may cause confusion and uncertainty by scientists and decision-makers in countries and globally. This paper aims to understand why discrepancies in estimates occur by elucidating the key differences in the way these entities estimate the number of global deaths due to nutrition-related risk factors such as preterm birth; stunting, wasting, and underweight in children; and suboptimal breastfeeding. We compare the statistical methods, inputs, and assumed links between inputs and outcomes used by major entities to estimate death and disability, and offer recommendations for improving estimates and presentation of methods. For IHME's GBD reports, we examine the methods through GBD 2015 since GBD 2016 was not published at the time of this analysis.

## Challenges in Estimating Global Disease Burden

**FIGURE 1:**
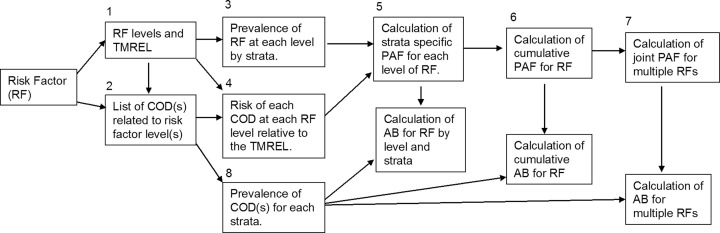
Conceptual model of the steps needed to estimate the GBD. AB, attributable burden; COD, cause(s) of death; GBD, Global Burden of Death study; PAF, population attributable fraction; TMREL, theoretical minimum risk exposure level.

Several specific steps are needed to estimate the global burden of disease due to a given risk factor (**Figure 1**). First, once a risk factor has been identified, the levels of the risk factor, which may be age dependent, are specified. For example, the categories of suboptimal breastfeeding are age dependent with nonexclusive breastfeeding applying from 0–6 mo and discontinued breastfeeding from 6–23 mo. The levels of breastfeeding for the period 0–6 mo are exclusive, predominant, partial, and none, whereas the levels for 6–23 mo are continued or discontinued breastfeeding. A particular risk factor level, or a combination of levels, will be used as the referent level. IHME refers to this level as the theoretical minimum risk exposure level (TMREL), or the lowest level of the risk factor that is plausible in a population. LiST includes the impact of the intervention, i.e., the distribution of the risk-factor levels after an intervention is employed. The TMREL and the impact of the intervention in LiST both play the role of setting the risk-factor distribution in a hypothetic healthy referent population. Commonly, the risk factor is simply not present in the referent population (e.g., all infants <6 mo old are exclusively breastfed), but at other times this level requires some analysis to determine what is the lowest level of the exposure one could realistically expect (e.g., 90% of infants <6 mo old are exclusively breastfed).

The second step is to link causes of death (CODs) to specific levels of a risk factor, e.g., linking suboptimal breastfeeding to deaths due to diarrhea and pneumonia among infants <1 y old. In the GBD 2013, 2015, and 2016, CODs were linked to risk factors based on the World Cancer Research Fund grades (17), where COD–risk-factor pairs are included with a grade of at least probable. As a result, “evidence strong enough to support a judgment of a probable causal relationship” (17) must be demonstrated for all COD–risk-factor pairs. Neither MCEE, LiST, nor LBS report global criteria for when a COD–risk-factor pair is linked. Instead these entities decide on linking CODs with risk factors based on rigorous scientific evidence (18). For anthropometry and breastfeeding, for example, all links were those supported by recent meta-analyses, and thus have strong evidence for linkage with death.

The third step is to estimate the prevalence of the risk factors for each country-year under consideration (commonly by age and sex as well). This is a complex modeling task, which requires a unified global model capable of accounting for study quality characteristics and nonlinear longitudinal patterns. The GBD usually implement a spatial-temporal Gaussian process regression (ST-GPR) or the DisMod-MR method (19, 20). The ST-GPR method is a 3-stage process that is used in a number of GBD publications (3, 4, 21, 22); it consists of removing systematic differences in the data sources, smoothing results across space and time, and implementing a GPR model for the final prevalence estimates. DisMod-MR is a hierarchical Bayesian procedure that corrects differences in data-collection methods, with a hierarchic spatial structure where years are nested within countries, regions, and super-regions.

MCEE used 2 separate prevalence estimation methods. One was developed by the Nutrition Impact Model Study Group (NIMS) (23) and the other is based on data from the joint child malnutrition estimates developed by UNICEF, WHO, and the World Bank (24), which is referred to as the UN method. The NIMS method is a hierarchical Bayesian model (25), whereas the UN method is a more straightforward multilevel mixed model. The prevalence of risk factors for LiST are drawn from the Demographic and Health Surveys (DHS), the Multiple Indicator Cluster Survey (MICS), and other nationally representative household surveys with no smoothing or modeling.

In the fourth step, the risk for each risk factor level relative to the referent level for all related CODs is estimated. For the risk factors considered here, this step is completed with the use of published meta-analyses, and there was considerable, but not total, overlap between the meta-analyses that were used by the different entities to estimate the RRs. The RRs used are unadjusted, except for stunting, wasting, and underweight in the GBD where a simulation method is used to estimate jointly adjusted RR values (discussed further in the next section). There are some CODs that are 100% attributed to a certain risk factor where a RR is not required. For the conditions studied here, deaths due to protein-energy malnutrition (PEM) are all attributed to wasting.

In step 5, once the RR and prevalence of a risk factor across all levels and strata are obtained, the population attributable fraction (PAF) can be estimated (26, 27). Standard formulas are used to calculate PAF, with the TMREL being used as the comparison group (26).

In steps 6 and 7, the burden attributable to multiple levels of a risk factor or multiple risk factors are estimated. This can be done assuming independence. For example, suppose the PAF for nonexclusive and discontinued breastfeeding is 20% and 10%, respectively. Then the burden attributable to both factors is 1 minus the proportion of children not impacted by either risk factor. In the example, this would be 28% [1 – (1 – 0.2) × (1 – 0.1) = 0.28]. The independence assumption has both biological and statistical components. First, the distributions of, say, factors A and B are assumed to be statistically independent, e.g., the prevalence of suboptimal breastfeeding is not related to the prevalence of discontinued breastfeeding. Second, the risk of an outcome given factors A and B is assumed to be equal to the product of the risk of the outcome given A and the risk of the outcome given B (i.e., $R{R_{AB}} = \ R{R_A}R{R_B}$) (28). Thus, the risk factors need to be statistically independent, and have etiologic influences that are biologically independent (29). In general, assuming independence will result in overestimation of the burden because positive dependence is likely present between the factors (30).

Because of the likely overestimation from the independence assumption, the GBD uses mediated-adjusted PAF when calculating the burden attributable to multiple risk factors. The mediated adjustments are made when combining risk-factor levels or multiple risk factors to a higher level of aggregation (e.g., combining stunting, wasting, and underweight into undernutrition). The adjustments are made by multiplying the PAF by a mediation factor, which estimates the proportion of the crude excess risk between a risk factor and an outcome that is mediated by other risk factors. Mediation factors are estimated with the use of a hypothesized pattern of association and data on the relationship between the factors [see pages 28–35 in the supplemental material of (4) for more detail]. The mediation-adjusted PAF will hypothetically adjust for overestimation due to the independence assumption, although the degree of adjustment or effectiveness has not been demonstrated in the literature. The GBD is the only entity to use mediation factors when combining risk factors.

For step 8, the number of deaths for each COD is estimated. The GBD 2015 uses a complex modeling algorithm that combines vital registration data, verbal autopsy surveys, and census data (22). The COD data are complicated by revisions in the International Classification of Diseases (ICD) (31), variation in garbage coding across countries and time, incomplete vital registration data, and large nonsampling variance among others. Garbage coding is an algorithm used to redistribute reported CODs that cannot or should not be the underlying COD (32). Attempts are made to attribute each death to a single underlying cause. Once the data have been assembled, they are smoothed with the Cause of Death Ensemble model (33). MCEE and LiST use the WHO cause-specific mortality estimates (34).

Common outcome metrics are the number of deaths, years of life lost (YLLs), years lived with disability (YLDs), and disability adjusted life years (DALYs, the sum of YLLs and YLDs). The focus here is on the number of deaths, since YLLs, YLDs, and DALYs are not reported by all groups. The total burden of a risk factor to an outcome (e.g., number of deaths due to diarrhea) is calculated by multiplying the PAF by the prevalence of the outcome. The total burden of a risk factor is calculated by summing its total burden over all associated outcomes (i.e., over all associated causes of death or disability).

## Comparing Estimates

### Stunting, wasting, and underweight in children

A summary of the assumptions and methods used by each entity is included in **Table 1**. The GBD 2015 study estimated the global impact of childhood undernutrition in terms of the total number of fatalities, YLLs, YLDs, and DALYs (4). Childhood undernutrition combines the effects of 3 undernutrition indicators: childhood stunting, wasting, and underweight. The joint and individual impact of these risk factors is reported. Combining the effects of stunting, wasting, and underweight is a difficult task since the RRs for each of these risk factors are not adjusted for the other 2 due to their high association. The GBD 2013 and 2015 studies accounted for the correlation between the undernutrition components by adjusting RRs with a simulation of the joint distribution of the conditions based on research by McDonald et al. (35). The adjusted RRs were then used to calculate the aggregated PAFs for stunting, wasting, and underweight.

The main inputs for the GBD 2015 undernutrition analyses were survey datasets [e.g., DHS, Reproductive and Health Surveys (RHS), MICS, Living Standards Measurement Surveys (LSMS)] along with tabulated datasets from survey reports or published articles extracted by the GBD [converted to the WHO child growth standards through the use of WHO algorithms (36) where appropriate].

**TABLE 1:** Summary of assumptions and methods made to produce global estimates of deaths due to undernutrition, underweight, stunting, or wasting in thousands for the GBD 2010 (2), 2013 (3), and 2015 (4) studies along with the MCEE (7, 8)^1^

Source	Year	Risk Factor	Levels	Prevalence Estimate	RR Estimate	TMREL	Linked COD	PAF Method	Input Data
GBD	2010	Underweight	Mild, regular, severe	ST-GPR^2^	Black et al. (2008) (7)	Proportion of the WHO 2006 reference population in each SD range	Intestinal infectious diseases, measles, malaria; the aggregate of LRIs, URIs, and otitis media; PEM	Assumes that risk factors are independent	Examination surveys and epidemiologic studies
GBD	2013	Stunting	Mild, regular, severe	ST-GPR	Olofin et al. (2013) (37)	All children aged <5 y above –1 SD HAZ	Diarrhea, LRIs, URIs, otitis media, measles	Simulation approach that accounts for covariance of all 3 risk factors based on McDonald et al. (2013) (35)	Examination surveys and epidemiologic studies
		Wasting	Mild, regular, severe	ST-GPR	Olofin et al. (2013) (37)	All children aged <5 y above –1SD WAZ	Diarrhea, LRIs, URIs, otitis media, measles, and PEM		
		Underweight	Mild, regular, severe	ST-GPR	Olofin et al. (2013) (37)	All children aged <5 y above –1SD WHZ	Diarrhea, LRIs, URIs, otitis media, and measles		
GBD	2015	Stunting	Mild, regular, severe	ST-GPR	Olofin et al. (2013) (37)	All children aged <5 y above –1SD HAZ	LRI, diarrhea, and measles	Simulation approach that accounts for covariance of all 3 risk factors based on McDonald et al. (2013) (35)	RHS, MICS, DHS, LSMS, CHNS, and others.
		Wasting	Mild, regular, severe	ST-GPR	Olofin et al. (2013) (37)	All children aged <5 y above –1SD WAZ	LRI, diarrhea, measles, and PEM		
		Underweight	Mild, regular, severe	ST-GPR	Olofin et al. (2013) (37)	All children aged <5 y above –1SD WHZ	LRIs, diarrhea, and measles		
MCEE	2004	Stunting	NS	UN	Black et al. (2008) (7)	NS	Diarrhea, pneumonia, measles, and malaria	Assumes that risk factors are independent	Joint UNICEF, WHO, and World Bank dataset
		Wasting	NS	UN	Black et al. (2008) (7)	NS	Diarrhea, PEM, pneumonia, measles, and malaria		
		Underweight	NS	UN	Black et al. (2008) (7)	NS	Diarrhea, PEM, pneumonia, measles, and malaria		
MCEE	2011	Stunting	NS	Both UN and NIMS	Olofin et al. (2013) (37)	NS	Diarrhea, pneumonia, measles, and other	Assumes that risk factors are independent	Joint UNICEF, WHO, and World Bank dataset
		Wasting	NS	Both UN and NIMS	Olofin et al. (2013) (37)	NS	Diarrhea, pneumonia, measles, and other		
		Underweight	NS	Both UN and NIMS	Olofin et al. (2013) (37)	NS	Diarrhea, pneumonia, measles, and other.		

1 CHNS, China Health and Nutrition Survey; COD, cause of death; DHS, Demographic and Health Survey; GBD, Global Burden of Disease study; HAZ, height-for-age *z* score; LRI, lower respiratory infection; LSMS, Living Standards Measurement Survey; MCEE, Maternal Child Epidemiology Estimation Group; MICS, Multiple Indicator Cluster Survey; NIMS, Nutrition Impact Model Study Group; NS, not specified; PAF, population-attributable fraction; PEM, protein-energy malnutrition; RHS, Reproductive and Health Survey; ST-GPR, spatial-temporal Gaussian process regression; TMREL, theoretical minimum risk exposure level; URI, upper respiratory infections; WAZ, weight-for-age *z* score; WHZ, weight-for-height *z* score.

2 This ST-GPR model is different to the latter methods and those discussed in detail in this paper.

To estimate the prevalence of childhood stunting, wasting, and underweight, the ST-GPR model was used on the country-level prevalence data. The covariates used were mean years of education among women of reproductive age, log-transformed lagged-distributed income, and total energy availability (kcal per capita). The prevalence of the undernutrition indicators was estimated in 3 separate categories: severe (<–3 SDs), moderate (from –3 to –2 SDs), and mild (from –2 to –1 SDs). The prevalence in each category was estimated for each age-sex group. The TMREL was no, mild, moderate, or severe stunting, wasting, or underweight.

The crude RRs for outcomes by each undernutrition indicator were obtained from a meta-analysis (37). As discussed above, the RRs were adjusted to account for covariance between the 3 undernutrition indicators via a simulation. The following outcomes were considered attributable to all undernutrition indicators: lower respiratory infections (LRIs), diarrhea, and measles. Deaths due to PEM were 100% attributed to childhood wasting, and thus no RR is required.

For MCEE the RRs for outcomes by each undernutrition indicator were obtained from the same meta-analysis as was used by GBD (37). All of the undernutrition indicators were associated with diarrhea, pneumonia, measles, and other infectious diseases (not including malaria). The final estimates are shown for both the UN and NIMS prevalence estimates of stunting, wasting, and underweight (8). The risk factors are not combined to obtain the impact of overall undernutrition.

#### Estimates of global burden

The GBD 2015 study estimated the total number of deaths (in thousands) due to childhood undernutrition (combining stunting, wasting, and underweight) to be 1265 [uncertainty interval (UI): 1160, 1383] in 2015 (see **Table 2**) (4). MCEE estimated the number of deaths for the period 0–23 mo in 2011 in low- and middle-income countries (LMICs) due to underweight as 999 and 1,180 (no UI for either) (8) depending on whether the UN (24) or NIMS (23) prevalence estimates were used, respectively.

**TABLE 2:** Global estimates of deaths due to undernutrition, underweight, stunting, or wasting in thousands for the GBD 2010 (2), 2013 (3), and 2015 (4) studies along with the MCEE (7, 8)^1^

Estimated deaths (in thousands) by year						
Report source and year	Risk factor	1990	2004/2005	2010/2011	2013	2015
GBD 2010	Undernutrition	2264		860		
GBD 2013	Undernutrition	3635			1327	
GBD 2013	Underweight	1080			386	
GBD 2013	Stunting	848			218	
GBD 2013	Wasting	3295			1247	
GBD 2015	Undernutrition		2093			1265
GBD 2015	Underweight		666			373
GBD 2015	Stunting		508			257
GBD 2015	Wasting		1882			1169
MCEE 2004/2011^2^	Underweight		1957	999/1180		
MCEE 2004/2011^2^	Stunting		1491	1017/1179		
MCEE 2004/2011^2^	Wasting		1505	875/800		

1 GBD, Global Burden of Disease; LMIC, low- and middle-income country; MCEE, Maternal Child Epidemiology Estimation Group; NIMS, Nutrition Impact Model Study Group.

2 Estimates for MCEE 2011 are for LMIC only (8) and using the UN (24) or NIMS (23) prevalence estimates, respectively.

#### Discussion of differences

The age groups of the deaths were slightly different between the studies. The GBD studies and MCEE 2004 included deaths of children <5 y old, whereas MCEE 2011 only considered deaths in the first 2 y of life.

Overall, the number of deaths attributable to each undernutrition indicator, and their combination, appears to be decreasing with time. There are likely many factors contributing to the decrease in deaths; it appears that the largest contributor was the decreasing prevalence of stunting, wasting, and underweight (4). Taking this trend into account, the GBD 2013 and 2015 studies show consistent estimates. Conversely, estimates for the attributable deaths in 1990 were ∼60% higher (from 2,264 to 3,635 thousand) from the GBD 2010–2013 studies.

The number of attributable deaths due to wasting for MCEE 2004 was 25% lower than the GBD 2005 estimate, but undernutrition and stunting estimates were higher for MCEE 2004 compared with GBD 2005. Overall, the estimated deaths due to stunting, wasting, and underweight show less variability for MCEE compared with those given by GBD.

The 2 separate statistical methods that MCEE used to estimate the prevalence of risk factors differ considerably in the types of data used and model complexity. The estimated attributable deaths obtained from each method were similar, however, suggesting that estimating the global attributable burden is robust to the statistical method used to estimate the prevalence of a risk factor. Furthermore, the estimation of the RR values used the same reference (37). Therefore, the differences in the individual burden of the undernutrition indicators appear to be due to the linked CODs. As noted above, MCEE links the same CODs to each undernutrition indicator (diarrhea, pneumonia, measles, and other infectious diseases not including malaria); GBD links 3 CODs to each undernutrition indicator (LRIs, diarrhea, and measles), whereas PEM is 100% attributed to wasting.

### Suboptimal breastfeeding

A summary of the assumptions and methods used by each entity is included in **Table 3**. The GBD 2015 study estimated the global impact of suboptimal breastfeeding in terms of the total number of fatalities, YLLs, YLDs, and DALYs (4). Suboptimal breastfeeding is broken into 2 separate risk factors: nonexclusive breastfeeding (children not exclusively breastfed if <6 mo of age) and discontinued breastfeeding (children who discontinue breastfeeding <2 y old). Similar to undernutrition, the datasets were obtained from surveys and tabulated data. The TMREL was exclusive breastfeeding in the first 6 mo and continued breastfeeding (any breast milk as a source of nourishment) from 6–23 mo.

The exposure distribution in the GBD 2015 study was obtained through the ST-GPR method. The GBD RR for each linked COD due to suboptimal breastfeeding was obtained from 2 published meta-analyses (no citation provided). The outcomes that were attributable to nonexclusive breastfeeding were diarrhea (in LMICs only) and LRIs. Discontinued breastfeeding was paired with diarrhea (in LMICs only).

The LBS (9) included similar suboptimal breastfeeding categories by country type (LMIC/non-LMIC). To estimate prevalence in these categories, data from systematic reviews of published studies, gray literature, and authors’ research data were used. Multilevel linear regression models were used to estimate linear trends in the indicators over time. LiST was used to predict how many deaths of children aged <5 y would be prevented if breastfeeding patterns were scaled up to near-universal optimal levels (see discussion below). LiST considers associations between suboptimal breastfeeding with diarrhea and pneumonia. The LBS added links to a number of other CODs (see Table3). The RRs for suboptimal breastfeeding and the CODs in children <5 y was determined via a recent meta-analysis (38). The LBS adjusted the RRs for neonatal other, other (1–59 mo), and neonatal prematurity to account for deaths not caused by suboptimal breastfeeding (e.g., deaths due to infectious diseases). The LiST data sources on early initiation, exclusive, and continued breastfeeding are DHS, MICS, and other nationally representative household surveys. The death rates due to the linked CODs are obtained from WHO cause-specific mortality estimates (39).

**TABLE 3:** Summary of assumptions and methods made to produce global estimates of deaths due to suboptimal breastfeeding in thousands for the GBD 2010 (2), 2013 (3), and 2015 (4) projects along with LBS (9) and the MCEE (7, 8)^1^

Source	Year	Risk Factor	Levels	Prevalence Estimate	TMREL	RR Estimation	Linked COD	PAF Method	Input Data
GBD	2010	Nonexclusive breastfeeding	Exclusive, predominant, partial, none	ST-GPR^2^	All children exclusively breastfed for first 6 mo	Black et al. (2008) (7) and Lamberti et al. (2011) (40)	Diarrhea; the aggregate of LRIs, URIs, and otitis media	Assumes that risk factors are independent	Population surveys
		Discontinued breastfeeding	Present/absent	ST-GPR^2^	Continued breastfeeding until 2 y	Black et al. (2008) (7) and Lamberti et al. (2011) (40)	Diarrhea		
GBD	2013^3^	Nonexclusive breastfeeding	Exclusive, predominant, partial, none	ST-GPR	All children exclusively breastfed for first 6 mo	Lamberti et al. (2013) (41)	Diarrhea and LRIs	Mediated adjusted RR's with independent PAF calculation	Largely population representative survey series such as DHS, MICS, LSMS, other national nutrition surveys, among others
		Discontinued breastfeeding	Present/absent	ST-GPR	Continued breastfeeding until 2 y	Lamberti et al. (2013) (41)	Diarrhea		
GBD	2015	Nonexclusive breastfeeding	Exclusive, predominant, partial, none	ST-GPR	All children exclusively breastfed for first 6 mo	Published meta-analyses (no citation)	Diarrhea (in LMICs only) and LRIs	Mediated adjusted RR's with independent PAF calculation	Micro data from surveys and tabulated data from scientific literature and reports (42).
		Discontinued breastfeeding	Present/absent	ST-GPR	Continued breastfeeding until 2 y	Published meta-analyses (no citation)	Diarrhea (in LMICs only)		
MCEE	2004	Nonexclusive breastfeeding	Exclusive, predominant, partial, none	NS	NS	Black et al. (2008) (7)	Diarrhea and pneumonia	Assumes that risk factors are independent	National survey data
		Discontinued breastfeeding	Present/absent	NS	NS	Black et al. (2008) (7)	Diarrhea and pneumonia		
MCEE	2011	Nonexclusive breastfeeding	Exclusive, predominant, partial, none	Both UN and NIMS	NS	Black et al. (2008) (7) and Lamberti et al. (2011, 2013) (40,41)	Diarrhea and pneumonia	Assumes that risk factors are independent	NS
		Discontinued breastfeeding	Present/absent	Both UN and NIMS	NS	Black et al. (2008) (7) and Lamberti et al. (2011, 2013) (40,41)	Diarrhea and pneumonia		
LBS	2015	Nonexclusive breastfeeding	Exclusive, predominant, partial, none	LiST	95% of children aged <1 mo and 90% of those <6 mo would be exclusively breastfed	Sankar et al. (2015) (38)	Diarrhea, pneumonia, neonatal sepsis, neonatal prematurity, and neonatal other (adjusted)	Assumes that risk factors are independent	DHS, MICS, and other nationally representative household surveys.
		Discontinued breastfeeding	Present/absent	LiST	90% of those aged 6–23 mo would be partly breastfed	Sankar et al. (2015) (38)	Diarrhea, pneumonia, meningitis, measles, malaria, pertussis, and other (adjusted)		

1 COD, cause of death; DHS, Demographic and Health Survey; GBD, Global Burden of Disease; LBS, Lancet Breastfeeding Series Group; LMIC, low- and middle-income country; LRI, lower respiratory infection; LSMS, Living Standards Measurement Survey; MCEE, Maternal Child Epidemiology Estimation Group; MICS, Multiple Indicator Cluster Survey; NIMS, Nutrition Impact Model Study Group; NS, not specified; PAF, population-attributable fraction; ST-GPR, spatial-temporal Gaussian process regression; TMREL, theoretical minimum risk exposure level; URI, upper respiratory infection.

^2^This ST-GPR model is different from the latter methods and those discussed in detail in this report.

3 In 2013 the GBD started modeling exclusive, predominant, and partial breastfeeding as proportions of any breastfeeding to ensure that the sum of these 3 types of feeding in children aged <6 mo does not exceed the total of all children receiving some breastfeeding in the same age group.

For MCEE (7, 8) the risk of morbidity and mortality from suboptimal breastfeeding in young children was estimated via a meta-analysis (7). Suboptimal breastfeeding was associated with death due to diarrhea or pneumonia. The UN and NIMS statistical methods to estimate the prevalence of suboptimal breastfeeding were used (8).

#### Estimates of global burden

In the GBD 2015 study, suboptimal breastfeeding was ranked as the 8th, 14th, and 22nd leading risk factor of mortality for 1990, 2005, and 2015, respectively (4). In the GBD 2010 study, it was ranked as the 5th and 14th leading risk factor for 1990 and 2010, respectively (2). GBD 2015 estimated the total number of fatalities due to suboptimal breastfeeding to be 391,000 (UI: 258,000, 550,000) in 2015 (**Table 4**) (4). In contrast, LBS found that near-universal breastfeeding could prevent 823,000 (no UI reported) annual deaths in children <5 y old for 2015 (9). MCEE estimated the number of deaths of children aged 0–23 mo in 2011 in LMICs due to suboptimal breastfeeding to be 804,000 (no UI) (8).

**TABLE 4:** Global estimates of deaths due to suboptimal breastfeeding in thousands for the GBD 2010 (2), 2013 (3) and 2015 (4) studies along with LBS (9) and the MCEE (7,8)^1^

		Estimated deaths (in thousands) by year				
Source and year	Breastfeeding practice	1990	2004/2005	2010/2011	2013	2015
GBD 2010	Suboptimal	1275		545		
GBD 2010	Nonexclusive	1118		476		
GBD 2010	Discontinued	157		69		
GBD 2013	Suboptimal	1344			501	
GBD 2013	Nonexclusive	1155			442	
GBD 2013	Discontinued	191			59	
GBD 2015	Suboptimal		592			391
GBD 2015	Nonexclusive		551			364
GBD 2015	Discontinued		55			37
LBS	Suboptimal					823
MCEE	Suboptimal		1251	804^2^		

1 GBD, Global Burden of Disease; LBS, Lancet Breastfeeding Series Group; LMIC, low- and middle-income country; MCEE, Maternal Child Epidemiology Estimation Group.

^2^Estimates are for LMICs and are for deaths in months 0–23 only (8).

#### Discussion of differences

LBS used a referent group (i.e., TMREL) that consisted of “95% of children younger than 1 month and 90% of those younger than 6 months would be exclusively breastfed, and that 90% of those aged 6–23 months would be partly breastfed” (9). In the GBD 2013 and 2015, the TMREL was exclusive breastfeeding until 6 mo and continued breastfeeding until 2 y (2,4). Thus, the comparison groups were similar, but LBS used a slightly more relaxed criterion because it allowed for some women to not follow optimal breastfeeding practices, whereas the GBD comparison group did not. The MCEE comparison groups were not explicitly mentioned (8), but were likely the same as GBD. The GBD, LBS, and MCEE 2004 included deaths of children <5 y old, and MCEE 2011 only considered deaths in the first 2 y of life.

The contribution of suboptimal breastfeeding to global disease burden was smaller for the GBD 2015 than for any of the other studies discussed here, including the GBD 2010. The GBD 2015 states that “for a subset of risks, minimal changes in exposure occurred between 1990 and 2015” which includes “non-exclusive and discontinued breastfeeding” (4). As a result, the exposure of children to suboptimal breastfeeding does not appear to be the reason for the relatively large decrease in the number of mortalities from GBD 2010 to GBD 2015. Furthermore, similar meta-analyses are used. Thus, the differences appear to be due to excluding upper respiratory infections, diarrhea in non-LMICs, and otitis media as linked CODs for nonexclusive breastfeeding in 2015 (whereas they were included in 2010).

There is a marked difference in the suboptimal breastfeeding estimates from GBD studies and those completed by LBS and MCEE, which found ∼2 and ∼1.5 times the number of deaths due to suboptimal breastfeeding than GBD, respectively. The RR values for nonexclusive (by breastfeeding pattern) and discontinued breastfeeding are contained in **Tables 5** and **6**, respectively. The groups differ by their definitions of infection (see Tables5 and 6), and thus the RR values have different interpretations. The cause-specific RR values for the GBD and MCEE were similar except for the risk of mortality due to LRIs, particularly for the no-breastfeeding group. For those >1 y old, the GBD did not link LRI deaths to suboptimal breastfeeding. The MCEE has an RR of 1.92 for discontinued breastfeeding and LRIs for 6 mo–2 y old. As a result, both GBD and MCEE linked LRI deaths to suboptimal breastfeeding for those <1 y old, but only MCEE linked these factors for 1–2 y old.

**TABLE 5:** Relative risks of deaths due to nonexclusive breastfeeding for the GBD 2015 (4) along with LBS (9) and the MCEE (8)^1^

	Overall			Diarrhea			Pneumonia/LRIs/Any Infection^2^		
	Predominant	Partial	None	Predominant	Partial	None	Predominant	Partial	None
GBD 2015^3^	NR	NR	NR	2.645	5.12	13.50	1.94	2.79	41.58
MCEE^4^	1.48	2.84	14.4	2.28	4.62	10.53	1.75	2.49	15.13
LBS^4^	1.48	2.84	14.4	NR	NR	NR	1.7	4.56	8.6

1 GBD, Global Burden of Disease; LBS, Lancet Breastfeeding Series Group; LRI, lower respiratory infection; MCEE, Maternal Child Epidemiology Estimation Group; NR, not reported.

2 The GBD used LRIs, the MCEE pneumonia, and the LBS any infections including sepsis, meningitis, pneumonia, diarrhea, measles, malaria, among others.

3 Relative risk for 7–27 d, the values for 28–364 d were similar.

4 Relative risk for the first 6 mo.

**TABLE 6:** Relative risks of death due to discontinued breastfeeding for the GBD 2015 (4) along with LBS (9) and the MCEE (8)^1^

	All	Diarrhea	Pneumonia	Any Infection^2^
GBD 2015^3^	NR	2.31	NL	NL
MCEE^4^	3.68	2.1	1.92	NL
LBS^4^	1.76^5^/1.97^6^	NR	NR	2.09^1^

1 GBD, Global Burden of Disease; LBS, Lancet Breastfeeding Series Group; LRI, lower respiratory infection; MCEE, Maternal Child Epidemiology Estimation Group; NR, not reported; NL, cause of death not linked.

^2^Death due to any infection including sepsis, meningitis, pneumonia, diarrhea, measles, malaria, among others.

3 Relative risk for 1–12 mo, the values for 1–4 y were similar.

4 Relative risk for 6 mo–2 y.

5 No breastfeeding for 6–11 mo.

6 No breastfeeding for 12–23 mo.

The total burden of suboptimal breastfeeding is an aggregate of the burden due to nonexclusive breastfeeding and discontinued breastfeeding. Thus, the differences in GBD estimates of the deaths due to suboptimal breastfeeding and those for LBS and MCEE are likely partially due to the differences in the aggregated PAF calculation (i.e., the mediation adjustment used by the GBD). The difference in the CODs linked to suboptimal breastfeeding, however, likely matters more. LBS linked diarrhea and pneumonia (0–59 mo), neonatal sepsis, neonatal prematurity (adjusted), and neonatal other (adjusted) along with meningitis, measles, malaria, pertussis, and other (adjusted) for 1–59 mo. MCEE linked diarrhea and pneumonia, whereas the GBD only linked diarrhea in LMIC regions and LRIs for those <1 y old only. The estimates that are the most difficult to reconcile are the burden estimates for suboptimal breastfeeding between MCEE and GBD. The papers linked the same outcomes (diarrhea and pneumonia), appeared to use the same meta-analysis (although GBD 2015 was missing the citation for theirs), and MCEE was implemented with 2 separate prevalence procedures (with similar results). The aggregated PAF calculation used by GBD 2015 will play some role, but the magnitude of the differences suggests there is some other factor. It is possible that the COD estimate plays a bigger role, though the method for this is not discussed explicitly by either study.

## Summary

Overall, the GBD reports less burden attributed to undernutrition and suboptimal breastfeeding than other groups. GBD deaths due to child undernutrition indicators were distributed differently than those produced by MCEE (Table 2). GBD attributed much of the burden to wasting, whereas MCEE has stunting, wasting, and underweight playing more equal parts. Furthermore, the number of deaths attributable to undernutrition by GBD was similar to the number of deaths attributable to each of underweight and stunting by MCEE. The LBS estimate of deaths due to suboptimal breastfeeding was 2.1 times larger than the GBD estimate for 2015 (Table 4); a similar discrepancy was seen for the GBD estimate of 2013 and the MCEE estimate of 2011.

The following factors, or some combination of them, could be the reason for different estimates produced by the studies: differences in data, prevalence of the risk factor, the COD prevalence estimation, RR values, the aggregated PAF calculation, and the CODs related to risk factors. MCEE reported attributable deaths for undernutrition indicators through the use of the UN and NIMS prevalence estimates (8). These methodologies have many differences, but the attributable burden values were similar. This suggests that the attributable burden is robust to the method used to estimate the prevalence of a risk factor. Further, the RR values come from the same meta-analyses in some cases. As a result, the differences in the individual burdens were likely due to the CODs linked to each of the risk factors. For undernutrition, MCEE linked the same CODs to each factor (diarrhea, pneumonia, measles, and other infectious diseases not including malaria); GBD linked 3 CODs to all factors (LRIs, diarrhea, and measles) whereas PEM was 100% attributed to wasting. For suboptimal breastfeeding, the LBS linked diarrhea and pneumonia (0–59 mo), neonatal sepsis, neonatal prematurity (adjusted), and neonatal other (adjusted) along with meningitis, measles, malaria, pertussis, and other (adjusted) for 1–59 mo. MCEE linked diarrhea and pneumonia, whereas the GBD only linked diarrhea in LMICs and LRIs for those <1 y. Furthermore, the total burden of suboptimal breastfeeding was an aggregate of the burden due to nonexclusive breastfeeding and discontinued breastfeeding. The larger attribution of deaths to suboptimal breastfeeding for LBS and MCEE was likely partially due to the differences in the aggregated PAF calculation (i.e., the mediation adjustment used by GBD). As a result, when reporting the attributable burden due to a risk factor that is split into multiple levels, the dependence between those levels should be considered.

The GBD statistical modeling techniques make many assumptions related to the underlying mechanisms. The statistical methods used in the GBD are complex, frequently modified, and primarily explained in extensive online supplemental documentation. The supplemental materials often cite previous versions of the GBD for technical details, and a definitive seed publication where the ideas are clearly spelled out is often lacking. Having the statistical methods used in GBD independently evaluated via peer-reviewed technical publications would clarify the properties of the methods [e.g., empirical coverage probabilities, which have been criticized (43)], the assumptions of the model techniques, and the appropriateness of the procedures [see (44) and (25) for examples of technical publications of global prevalence estimates]. A benefit of the GBD methods is that the estimates of the number of attributable deaths are reported with UIs. Neither of the MCEE or LBS publications report UIs with their attributable burden estimates. UIs are important to accurately reflect the variability in estimated quantities to avoid conclusions that may have a large degree of uncertainty in them.

The estimates produced as part of the GBD, MCEE, and LBS have a large impact on nutrition policy decisions for global health initiatives. The power of these estimates necessitates the need for transparency in the methods used to produce the estimates by all entities. Transparency in the assumptions made during data cleaning and statistical modeling (via technical publications) increases the ability of other researchers to understand their weaknesses and strengths. In this paper, we found that the CODs linked to a risk factor are likely the biggest driver of the differences in the estimates. In the future, the attribution of different CODs to the overall burden estimates should be unpacked with the use of sensitivity analyses, especially when CODs are removed or added from previous work. These sensitivity analyses will clarify if differences in estimates, say from one GBD report to another, are due to differences in the modeling assumptions. The comparisons made in this paper are possible because different groups are producing estimates of global burden. Having groups that use contrasting modeling strategies to produce estimates benefits the nutrition community by showing the impact of different sets of assumptions and methods. Producing estimates of global burden is a difficult task requiring complex modeling. When multiple entities produce estimates and are transparent in their describing assumptions and methods, the mutually beneficial discussion and collaboration fostered will lead to improved global health monitoring.

## Notes

This research was supported by the Bill & Melinda Gates Foundation.

Author disclosures: EGP and the spouse of SYH work for the Bill & Melinda Gates Foundation.

Abbreviations used: COD, cause of death; DALY, disability-adjusted life-year; DHS, Demographic and Health Surveys; GBD, Global Burden of Disease study; GPR, Gaussian process regression; ICD, International Classification of Diseases; IHME, Institute for Health Metrics and Evaluation; LBS, Lancet Breastfeeding Series Group; LiST, Lives Saved Tool; LMIC, low- and middle-income country; LRI, lower respiratory infection; LSMS, Living Standards Measurement Surveys; MCEE, Maternal Child Epidemiology Estimation Group; MICS, Multiple Indicator Cluster Survey; NIMS, Nutrition Impact Model Study Group; PAF, population-attributable fraction; PEM, protein-energy malnutrition; RHS, Reproductive and Health Surveys; ST-GPR, spatial-temporal Gaussian process regression; TMREL, theoretical minimum risk exposure level; UI, uncertainty interval; YLLs, years of life lost; YLDs, years lived with disability.
